# Cancer Burden Among Arab World Males in 2020: The Need for a Better Approach to Improve Outcome

**DOI:** 10.1200/GO.21.00407

**Published:** 2022-03-30

**Authors:** Layth Mula-Hussain, Hala Mahdi, Zhian Salah Ramzi, Marwan Tolba, Mohamad Saad Zaghloul, Zineb Benbrahim, Atlal Abusanad, Humaid Al-Shamsi, Adda Bounedjar, Abdul-Rahman Jazieh

**Affiliations:** ^1^ Sultan Qaboos Comprehensive Cancer Care and Research Centre, Muscat, Oman; ^2^ Faculty of Health Sciences, McMaster University, Ontario, Canada; ^3^ College of Nursing, University of Sulaimani, Sulaimani, Kurdistan, Iraq; ^4^ McGill University, Montreal, Quebec, Canada; ^5^ Faculty of Medicine, Cairo University, Cairo, Egypt; ^6^ Faculty of Medicine and Pharmacy Fez, Université Sidi Mohamed Ben Abdellah, Fez, Morocco; ^7^ Faculty of Medicine, King Abdulaziz University, Jeddah, Kingdom of Saudi Arabia; ^8^ Burjeel Cancer Institute, Burjeel Medical City, Abu Dhabi, United Arab Emirates; ^9^ Emirates Oncology Society, Dubai, United Arab Emirates; ^10^ College of Medicine, University of Sharjah, Sharjah, United Arab Emirates; ^11^ Laboratoire de cancérologie, Faculté de Médecine, Université Blida 1, Blida, Algeria; ^12^ Cincinnati Cancer Advisors, Cincinnati, OH

## Abstract

Cancer is a leading cause of increased morbidity and mortality worldwide. This work aims to study the Arab world males' cancers (AMCs) and the similarities and disparities with the world males' cancers (WMCs) from different burden points of view.

**MATERIALS AND METHODS:**

A descriptive review of the 2020 Global Cancer Observatory revealed AMCs compared with the 2020 WMCs and the 2018 AMCs. Data on the top 27 AMCs were compared among the region's countries and the world groups.

**RESULTS:**

In 2020, a total estimate of 217,203 new AMCs, 2.2% of WMCs, with an average age-standardized rate of 133.5/100,000 population, compared with 222/100,000 population of WMCs, was observed. Death estimates were 148,395, 2.7% of WMCs, with an average age-standardized rate of 95/100,000 population, compared with 120.8/100,000 population of WMCs. The five-year prevalence was observed in 442,014, 1.8% of WMCs. The average AMC mortality to incidence ratio (MIR) was 0.68, compared with 0.55 in WMCs and 0.54 in Arab females. Lung cancer was the top in incidence and mortality, whereas penile cancer was the lowest. The range of MIRs among the 27 cancer types was 0.19-0.96.

**CONCLUSION:**

The descriptive review of the 2020 males' cancers in the Arab world revealed a relatively high MIR, compared with males' cancers worldwide and the females' cancers in the Arab world. This requires further evaluation to discern the underlying causes and address them systematically. More cancer control actions are warranted.

## INTRODUCTION

Cancer is one of the top three causes of death in most of the world's countries.^1^ One in five men and one in six women worldwide develop cancer during their lifetime, and one in eight men and one in 11 women die from the disease.^2^ In 2020, it was estimated that there were 50,550,287 patients with cancer worldwide, with 19,292,789 patients with new cancer and 9,958,133 new cancer deaths.^3^ The cancer incidence and mortality burden will be doubled by 2040.^4^

**CONTEXT**

**Key Objective**
What is the status of the males' cancers in the Arab world countries? Is there any difference compared with the position of cancer in the Arab world females or that in the males globally?
**Knowledge Generated**
Compared with Arab females, Arab males showed a lower incidence, higher mortality, lower prevalence, and higher mortality to incidence ratio. Compared with worldwide males, the incidence, mortality, and prevalence were lower, but the average mortality to incidence ratio was higher. Still, it is comparable with the ratio in the medium human development index and lower-middle–income countries.
**Relevance**
Cancer seems to be of greater burden in Arab males than Arab females and worldwide males. Further in-depth studies are warranted. More attention is required to decrease cancer incidence and improve the management outcomes in Arab males' cancers.

Globally, males constitute about 50.4% of the world population (3,929,973,836 of the total 7,794,798,844 as in the 2020 estimate). However, it seems that the burden of cancer on males is greater compared with females. The 2020 Globocan registry showed that male patients with cancer worldwide accounted for 52.2% of the total new cases and 55.5% of the deaths. At the same time, they were 49.1% among the prevalent cancer cases,^3^ which may indicate that cancer in males, compared with females, might be more aggressive and less curable.

Although the cancer burden is expanding in countries with different income levels because of the growth and aging of the population, about 70% of cancer deaths occur in low- and middle-income countries (LMICs).^5^ Cancer is usually diagnosed at an advanced stage in these less-fortunate nations, where modern diagnoses/treatments might be inaccessible. Compared with more than 90% of treatment availability in high-income countries (HICs), < 15% is available in low-income countries (LICs).^6^

The number of countries that are members of the League of Arab States is 22. They spread out across the Middle East and North Africa region, with more than 436 million, 51.8% of that being males (as shown in Table 1). Although these countries share historical, geopolitical, social, and cultural characteristics, the economy, human resources, and development vary widely across the region. The Arab countries are classified into three categories according to their gross national income: LICs, including Comoros, Djibouti, Mauritania, Yemen, and Somalia; middle-income countries, including Algeria, Egypt, Iraq, Jordan, Lebanon, Libya, Morocco, Palestine, Sudan, Syria, and Tunisia; and HICs, including Bahrain, Kuwait, Oman, Qatar, the Kingdom of Saudi Arabia (KSA), and the United Arab Emirates (UAE).^7^ The available literature indicates that cancer incidence rates are rising in Arab countries, meaning that its burden will be immense in the future.^8,9^

**TABLE 1:**
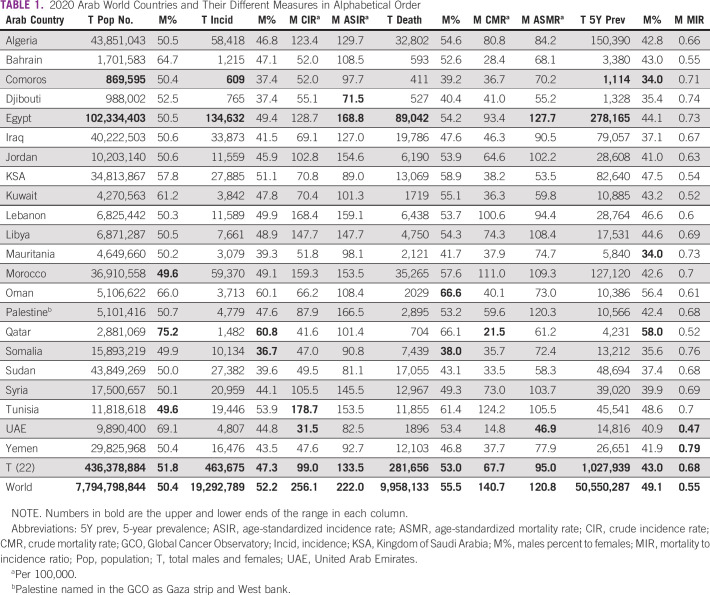
2020 Arab World Countries and Their Different Measures in Alphabetical Order

Literature on males' cancers in the Arab world is collectively scarce, and this group of patients may need more attention to improve care outcomes. In this work, we are aiming to describe all males' cancers in the Arabic countries (abbreviated as Arab world males' cancers—AMCs) from different epidemiologic perspectives, such as incidence, mortality, prevalence, and mortality to incidence ratio (MIR), in all the Arabic countries and the top 27 identified cancers. In addition, we explored some of the comparisons in AMCs between the Arabic countries themselves and world males' cancers (WMCs), including the world groups of countries that share specific human development index and income levels.

## MATERIALS AND METHODS

The 2020 Global Cancer Observatory (GCO), an international cancer database, was used as the primary source of the findings of this work.^3^ A literature search was conducted on May 17, 2020, via PubMed medical search engine using the following words: (“Male”[MeSH] AND “Arabs/statistics and numerical data”[MeSH] AND “Arabs/epidemiology”[MeSH] AND “Neoplasms”[MeSH]), with 13 results, and none of them was dealing with the subject of this work. Google Scholar was also used, but the results were mostly country- and cancer-specific and did not comprehensively cover the AMCs spectrum. The crude incidence rate, the age-standardized incidence rate (ASIR), the crude mortality rate, the age-standardized mortality rate (ASMR), and the prevalence rates were taken from the GCO. This was possible by entering the names of the Arab world countries to extract these rates in the males' cancers. We compiled and calculated the MIR for each country and cancer type accordingly. MIRs for the cancers, countries, and world groups were calculated by dividing the mortality cases over the incident cases. Using the GCO again, we compared the 2020 males' cancer statistics with those in 2018. In addition, we calculated and compared the male to female ratios for the 2020 population, cancer incidence, mortality, prevalence, and MIR in the Arabic countries and with the global measures. Simple descriptive statistics were used to calculate the frequencies and percentages. The Microsoft Excel for Mac (version 16.55) was used for the MIR calculations and to calculate other related measures and findings in this project.

## RESULTS

In 2020, statistics showed that the population of the countries of the Arab world was estimated to be 436,378,884 in total, corresponding to 5.6% of the total global population. Furthermore, 51.8% of the Arab world population in 2020 was males (range 49.6-75.2), and females constituted the remaining 48.2%. New cancer cases were 463,675 in total, corresponding to 2.4% of the global incident cases, and males' cancers accounted for 47.3% of that number, compared with 52.7% globally. The ASIR for males was 133.5/100,000 population, compared with 222/100,000 population in WMCs. The deaths were 281,656 in total, corresponding to 2.8% of the global deaths, and males' deaths were 53% of that total, compared with 55.5% globally. The male ASMR was 95/100,000 population, compared with 120.8/100,000 population in WMCs. The 5-year prevalent cases were 1,027,939, corresponding to 2% of the overall global survivors, and the Arab males were 43%, compared with 49.1 globally. The MIR average was 0.68 (compared with 0.55 globally), ranging from 0.47 to 0.79. More details are given in Table 1.

Compared with 2018, the population of Arab countries in 2020 increased by 3% (from 422,717,439 to 436,378,884) and the males' proportion decreased from 54.3% to 51.8%. However, males' proportion of the new cancer cases increased from 46% to 47.3%, their proportion of total deaths increased from 52% to 53%, and their proportion of prevalent cases increased from 41.4% to 43%. Males' ASIR increased from 127.4 to 133.5/100,000 population, and the ASMR also increased from 91 to 95. MIR improved in AMCs from 0.69 to 0.68 and globally from 0.57 to 0.55. Tables 1 and 2 include 2020 and 2018 details.

**TABLE 2:**
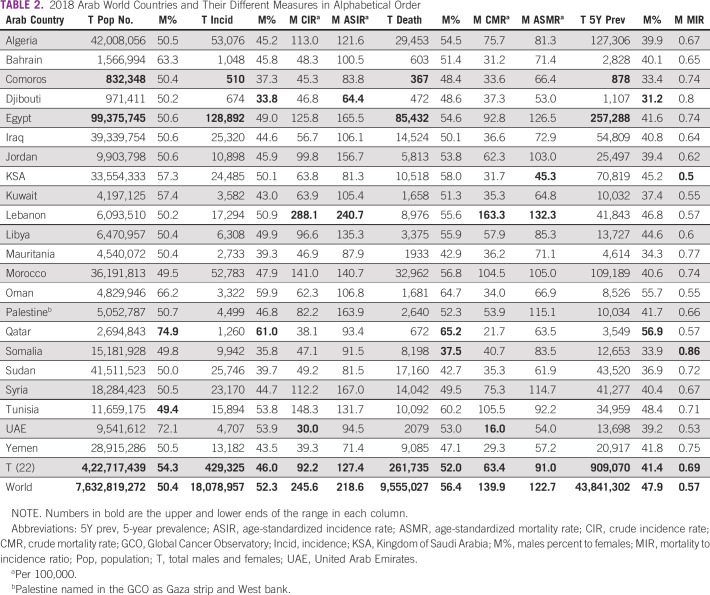
2018 Arab World Countries and Their Different Measures in Alphabetical Order

The 27 cancers included in the AMC category in 2020 and 2018 are detailed in Tables 3 and 4. Lung cancer was topping the list as the highest cancer in incidence, crude incidence rate, ASIR, deaths, crude mortality rate, and ASMR. By contrast, penile cancer was the lowest in the measures mentioned above. The lowest MIR was in thyroid cancer with 0.19, and the highest was in esophageal and pancreatic cancers with 0.96. Compared with 2018, the 27 cancer types in Arab country males in 2020 increased by 7.6% (from 201,831 to 217,203) and their deaths increased by 6.9% (from 138,832 to 148,395). Males' MIR improved from 0.69 to 0.68. The most incident cancer type, the lung, improved in terms of MIR from 0.97 to 0.90. The most unfavorable cancers in 2018, the liver and the pancreas, improved in MIR from 0.98 both to 0.95 and 0.96, respectively.

**TABLE 3:**
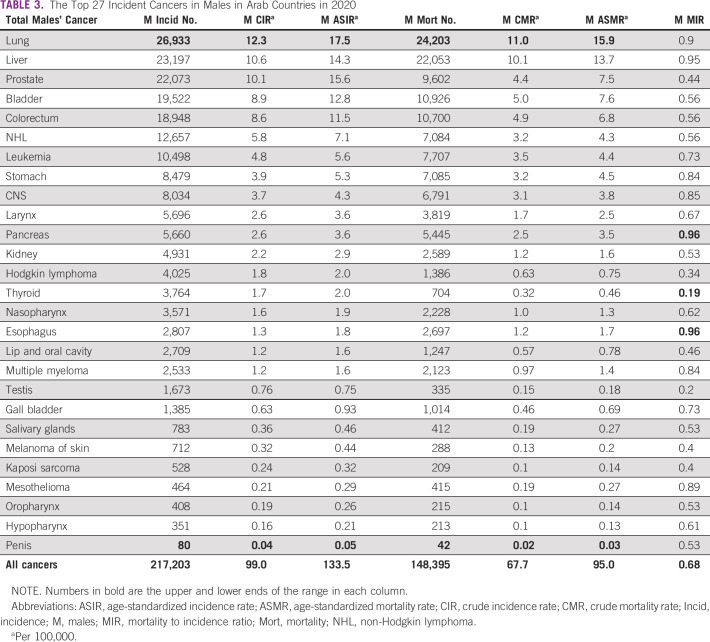
The Top 27 Incident Cancers in Males in Arab Countries in 2020

**TABLE 4:**
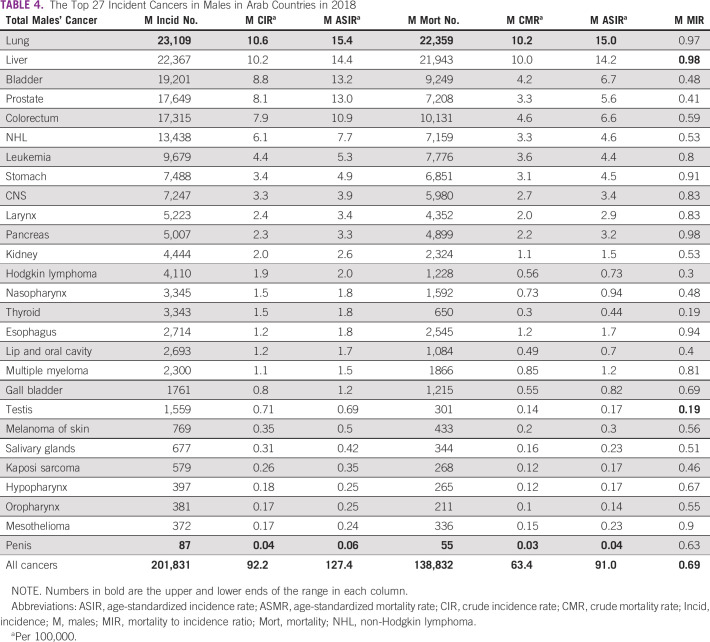
The Top 27 Incident Cancers in Males in Arab Countries in 2018

Upon comparing the 2020 top 10 cancers between AMC and WMC, we recognize that eight cancers are common in the incidence list (lung, liver, prostate, bladder, colorectum, non-Hodgkin lymphoma, leukemia, and stomach) with variation in the sequence of these eight cancers. CNS and laryngeal cancers were among the list in the AMC, whereas esophageal and kidney cancers were in the list of WMC. The mortality list revealed that nine cancers are common (lung, liver, bladder, colorectum, prostate, leukemia, stomach, non-Hodgkin lymphoma, and pancreas), and CNS and esophageal cancers were the nonshared deadly cancers in the top 10 of the AMC and WMC, respectively. Table 5 shows the complete list.

**TABLE 5:**
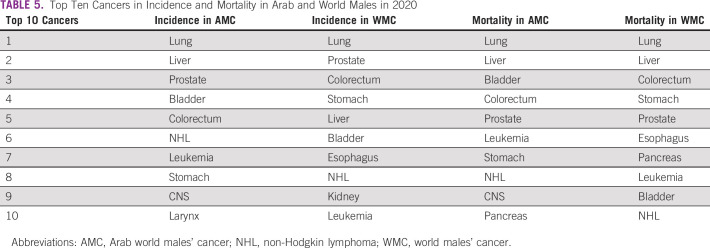
Top Ten Cancers in Incidence and Mortality in Arab and World Males in 2020

The comparison between the AMC and other human groups showed that the MIR is 0.68 in AMC and 0.55 in WMC. World countries are classified on the basis of the human development index (HDI) into four categories^3^: very high HDI, high HDI, medium HDI, and low HDI. At the same time, world countries are classified on the basis of income level into another four categories^3^: HICs, upper-middle–income countries, LMIC, and LICs. From Table 6, MIR in AMC (0.68) seems to be in proximity to that in medium HDI (0.69) and to that in LMICs (0.69). Table 6 shows the details.

**TABLE 6:**
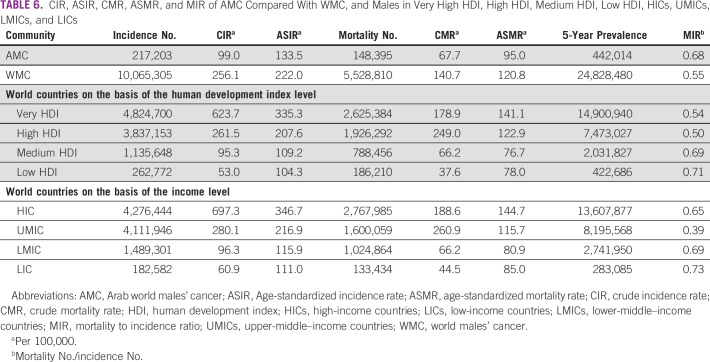
CIR, ASIR, CMR, ASMR, and MIR of AMC Compared With WMC, and Males in Very High HDI, High HDI, Medium HDI, Low HDI, HICs, UMICs, LMICs, and LICs

## DISCUSSION

Our study showed that in 2020, males constituted 51.8% of the Arab world population, 47.3% of the patients with new cancer, 53% of the cancer deaths, and 43% of the 5-year cancer prevalence. The average MIR was 0.68, compared with 0.55 worldwide. The lowest MIR was in thyroid cancer (0.19), and the highest was in esophageal and pancreatic cancers (0.96). Lung cancer ranked the highest in incidence and death numbers, followed by liver, prostate, bladder, and colorectum in incidence and liver, bladder, colorectum, and prostate in deaths. Penile cancer was the lowest prevalent cancer type.

This study on the AWCs' cancers is in sequence with previous work on the Arab world females' cancers on the basis of the 2018 and 2020 GCO estimates.^8,10^ In comparing the MIR between males and females in the Arab world and globally, we find that MIR is favorable in females than males in the two cohorts. It was 0.54 in 2018 and remained stable in 2020 for the females in the Arab world. It slightly improved from 0.49 to 0.48 globally. This is comparable with males in the Arab world, 0.69 to 0.68, and worldwide, 0.57 to 0.55, in 2018 and 2020, respectively. Although the males' ratio is 51.8% of the population, their incidence percentage is 47.3%, their deaths are 53%, and their prevalence is 43%. This indicates that cancers in males are more aggressive and less curable than those in females in the Arab world. Moreover, the gap of the MIR between the Arab world and the global indices is more prominent in males than in females, as it is 0.13 compared with 0.06 in 2020. This highlights the importance of paying more attention to the prevention and care outcomes in males' cancers.

MIR of cancers among AWCs showed modest improvement from 2018 (0.69) to 2020 (0.68). The MIR of cancer among Arab world females was stable in 2018 and 2020 at 0.54. Globally, the MIR improved from 0.57 in 2018 to 0.55 in 2020 in males and from 0.49 in 2018 to 0.48 in 2020 in females.^8,10^ This favorable outcome in females' cancers compared with males in the Arab world and globally can be attributed to a large extent to the relatively good outcomes to the most common cancer in the females, which is breast cancer, and the early detection programs and the advanced therapies that led to the prevention of death in many of the females. High smoking rates in males in the Arab world, compared with females, with low use of lung screening in the region, might contribute more to this difference. More public awareness and media attention toward females' cancers than males might also be a factor.

Aside from the males' and females' statistics, there were several prominent differences between the data within Arab countries and worldwide data. The ASIR and ASMR for AWCs versus global males are 133.5/100,000 versus 222/100,000 and 95/100,000 versus 120.8/100,000, respectively. The low cancer rates can be attributed to lower risk factors, a younger population, fewer diagnostic facilities, and suboptimal registry data. However, these low incidence and death rates were not consistent with MIR between the two cohorts, as the average value for Arab countries was 0.68, whereas the global average was 0.55. The higher MIR can be related to late diagnosis, lack of screening programs, and lack of access to best treatment because of many reasons, such as high cost and inadequate human resources. These trends in regional and worldwide data indicate that although males in Arab countries have lower incidence and mortality rates than males globally, they generally have less favorable outcomes, as seen from the higher MIR.

Upon comparison of 2018 and 2020 data sets, it is interesting to find that although the percentage of males in the Arab world population decreased from 54.3% to 51.8%, the total number of new cases, deaths, and prevalent cases all increased from 46% to 47.3%, 52% to 53%, and 41.4% to 43%, respectively. Other notable changes are that the male ASIR increased from 127.4 per 100,000 in 2018 to 133.5 per 100,000 and the male ASMR also increased from 91 per 100,000 to 95 per 100,000. However, the MIR did improve from 0.69 in 2018 to 0.68 in 2020, which indicates a slight improvement in the outcome of male cancers in the Arab world.

Although the average male to female ratio in the Arab world population in 2020 is 51.8:48.2 (compared with the worldwide ratio of 50.4:49.6), it is almost equally distributed (around 51% ± 1.4) in most Arab countries. However, males are more in Gulf countries (57.8% in Saudi Arabia, 61.2% in Kuwait, 64.7% in Bahrain, 66% in Oman, 69.1% in UAE, and 75.2% in Qatar, Table 1). The main reason for this abnormal ratio in the Gulf region countries is the large male migrant stock.^8^

Upon examining the results, we notice that there is a modest improvement in most aggressive cancers' MIR between 2018 and 2020, such as liver (0.98-0.95), lung (0.97-0.9), mesothelioma (0.9-0.89), pancreas (0.98-0.96), and stomach (0.91-0.84), excluding the esophagus (0.94-0.96). These cancers represent a great challenge and have the highest MIR (their average is 0.92). This modest improvement might reflect the advances in cancer management and recent treatments such as new immunotherapy lines, new surgical techniques with increased numbers of well-trained staff, and the adoption of new precise radiotherapy techniques in different Arab countries, for example, stereotactic and image-guided radiotherapy.^11^

Lung cancer is the top in incidence and deaths with a MIR of 0.9. In this regard, despite the global awareness to control tobacco and the low prevalence of tobacco use among males in some Arab countries, like Oman, with 19.2% in 2020, the data from other countries are alarming. Tobacco use, cigarettes, or other types like shisha (Argela or waterpipe) increase in some Arab countries' males.^12^ Males' tobacco use in 2020 was at 49.5% in Lebanon, 47.2% in Tunisia, 42.2% in Egypt, 42% in Bahrain, and 41.5% in Kuwait. The estimates in these countries in 2025 will be at 50.2%, 42.2%, 42.6%, 42.3%, and 40%, in sequence.^13^ This, unfortunately, will turn into more tobacco-related cancers in the future.

It is worth mentioning that the MIR from 2018 to 2020 had changed favorably and unfavorably in some Arab countries. In Iraq, Palestine, Libya, Syria, and Yemen, the MIR unfavorably increased from 0.64, 0.66, 0.60, 0.67, and 0.75 to 0.67, 0.68, 0.69, 0.69, and 0.79 (highest MIR in AWMs), in sequence. On the other hand, the MIR favorably decreased in Morocco, Bahrain, Qatar, Kuwait, and UAE from 0.74, 0.65, 0.57, 0.55, and 0.53 to 0.70, 0.55, 0.52, 0.52, and 0.47 (lowest MIR in AWMs), in sequence. These changes might probably be attributed to the disparities in health care services between the Arab countries, HDI, income level, and war-torn national status in some Arab nations.^14-16^

Notably, liver cancer comes second in incidence (with 23,197) and deaths (with 22,053) after lung cancer (with 26,933 and 24,203, respectively) in the AMCs. However, it is not that common in many individual Arab countries. This is attributed to the high burden and prevalence of liver cancer in a single large-populated nation, Egypt, where the population is more than 102 million, about a quarter of the Arab world population. Among the total liver cases in AMCs in 2020, Egypt constituted 78% of the incidence and deaths of liver in AMCs (18,145 and 17,211, respectively). Liver cancer in Egypt ranked first in 2020 (27,895, 20.7%, even before the breast cancer that came with 22,038, 16.4%). Egypt is still and has been suffering for years from a high burden of hepatitis, the biggest contributing risk factor in developing liver cancer.^17^ As Egypt has the highest prevalence of hepatitis C virus infection globally, the health authorities there started in 2018 a nationwide screening and treatment campaign that covered about 50 million. It successfully provided free treatment for all Egyptian patients with hepatitis C virus infection.^18^ Hopefully, this step will be projected as a decrease in the incidence and an improvement in the outcome of this aggressive cancer in the coming years.

Although prostate cancer is either the first or second highest in many American and European countries, it is third in AMCs, after the lung and liver, because of differences in aging, lifestyle, and screening programs. Besides other cancers in the top 10 list mentioned above, the CNS tumors are in the AMCs list and not in the WMCs list. The CNS tumors were sixth in 2020 in Iraq and used to be fourth in 2010, which might be attributed to war-related environmental effects that the country has been through (CNS was ranked eighth in 1998).^19^

Comparing the statistical trends with other world groups, we notice that the MIR of 0.68 in AMCs is close to that of countries with the medium HDI (with a MIR of 0.69) and LMICs (with a MIR of 0.69). We consider this comparison reasonable as just six countries are HICs, and their population is about 13.5% of the total Arab world population. By contrast, 86.5% live in 16 other nations with middle- and low-income levels.

These trends of AMCs necessitate extensive and in-depth analysis for more actions, as some data further support duplication of the burden of cancer in the Middle Eastern region over the next decade.^20^ Although 80% of countries in the region have national cancer control policies, only 45% of these programs are operational.^21^ We think that increasing the attention toward the cancer control policies and services is part of the cancer patients' rights campaign that needs to be fulfilled.^22^ In addition, the total research output remains low,^23^ particularly in studies related to preventative cancer control policies.^21^ On the basis of all the above, more attention toward improving the cancer care services is required.^24^

The strengths of this work come from the recent estimates of the cancer statistics, the multinational Arab collaboration, and input from different countries and across the world, being the first collective review concerning AMCs as a spectrum, compared with Arab females' cancers and worldwide males. Also, it can be a cornerstone for the following in-depth studies and analyses. Regarding the limitations of this work, mainly, it is a descriptive study and based on an estimate from another comprehensive global database. Other limitations can include the scarce resources and lack of robust data, reporting, and documentation in the Arab countries' cancer registries.

In conclusion, the descriptive review of the 2020 males' cancers in the Arab world revealed a relatively high MIR, compared with males' cancers worldwide and the females' cancers in the Arab world. This requires further evaluation to discern the underlying causes and address them systematically. More cancer control actions are warranted.
